# Prevalence of needlestick and sharps injuries in the healthcare workers of Iranian hospitals: an updated meta-analysis

**DOI:** 10.1186/s12199-018-0734-z

**Published:** 2018-09-07

**Authors:** Reza Ghanei Gheshlagh, Marzieh Aslani, Fidan Shabani, Sahar Dalvand, Naser Parizad

**Affiliations:** 1 Clinical Care Research Center, Kurdistan University of Medical Sciences, Sanandaj, Iran; 2 Shahid Beheshti Hospital of Hamadan, Hamadan University of Medical Sciences, Hamadan, Iran; 3 Department of Nursing, University of Social Welfare and Rehabilitation Sciences, Tehran, Iran; 4 MSc of Biostatistics, Health Promotion Research Center, Iran University of Medical Sciences, Tehran, Iran; 5 Department of Medical-Surgical Nursing, School of Nursing and Midwifery, Urmia University of Medical Sciences, Pardis Nazlou, 11 km of Nazlou Road, Urmia, Iran

**Keywords:** Health care workers, Iran, Meta-analysis, Needlestick, Prevalence

## Abstract

**Background:**

Needlestick and sharps injuries (NSIs) are critical occupational risk among health care workers (HCWs), which is extremely worrying due to the potential risk of transmitting bloodborn pathogens (BBPs). This study was carried out to evaluate the prevalence of NSIs among Iranian HCWs.

**Methods:**

In this systematic review and meta-analysis, the key terms percu* injur*, needle* stick injur*, needlestick* injur*, or sharp* injur* were searched in the Scientific Information Database (SID), MagIran, IranMedex, Google Scholar, Science Direct, PubMed, and Scopus. A prefabricated checklist, including variables: first author, publication year, study population, sample size, gender, total prevalence of needlestick in each gender, type of questionnaire, region, and type of hospitals, was used to extract data from the selected articles included which were published between 2003 and 2016.

**Results:**

The analysis showed that the prevalence of NSIs in the Iranian HCWs was 42.5% (95% CI 37–48). Moreover, the prevalence of NSIs was more in women (47%; 95% CI 36–58) compared to men (42%; 95% CI 26–58).

**Conclusion:**

Given the high prevalence of NSIs, it is necessary to supply safe needles and instruments, hold training programs focused on new methods of using sharp objects safely, observe safety principles and standards, reinforce the practical skills of personnel, and pay more attention to reporting and improving occupational behaviors like avoiding needle recapping in order to reduce the prevalence of NSIs and consequently reduce potential risk of transmission of BBPs.

## Background

Healthcare workers are at greater risk of occupational exposure to splashes, sharps, and needlestick injuries (SSNIs) [1]. Since, splashing of blood and body secretion has not been mentioned in the majority of studies and their focus was on needlestick and sharp injuries in Iran. Therefore, the researchers investigated NSIs and splashed in the eyes with blood, and body secretions were excluded in this study. Needlestick and sharps injuries are impairments caused by needlestick, a piece of broken ampule, cannula, surgical blade, or other sharp instruments contaminated with blood or body secretions [2]. In 2008, more than 35 million HCWs around the world were exposed to NSIs [3]. In the USA, about 600,000 to 1 million NSIs occur per year, half of them are not reported [4]. Nowadays, NSIs are a serious work-related hazard and a potential risk of transmission of BBPs [5]. Following NSIs, around 20 types of pathogens can be transferred through blood, which is always worrying due to the potential risk of transmission of BBPs [6]. Worldwide, around 40% of HCWs suffer from hepatitis B and C virus infection and 2.5% are affected by human immunodeficiency virus (HIV) caused by NSIs [7]. These injuries not only raise the possibility of negative health consequences, but also lead to psychological distress, fear, tension, and anxiety in HCWs which results in increasing absence from work and have a direct negative effect on the health care services [8, 9]. On the other hand, medical treatment, blood work, and missed days at work for these injured individuals impose a high cost on the health care system [10]. Although more than 80% of NSIs can be prevented by observing standard precautions, NSIs are on the rise due to lack of adherence to standard infection control precautions on management and disposal of garbage and clinical waste [11].

Various studies have reported the prevalence of NSIs to be 68% in Jordan [12], 74% in South Korea [13], and 30% in Turkey [14]. Although the reporting of NSIs is important for prevention and treatment, but about 59% of HCWs do not report their injuries in Iran [15]. The degree of under-reporting of NSIs in HCWs may be as much as ten-fold. Hence, the health care authorities should not interpret this low prevalence rate as less injury in HCWs [16]. This is a very important issue; however, there are few studies conducted to determine prevalence in Iran. Knowing the latest statistics about the prevalence of NSIs could be helpful in designing and implementing programs and guidelines to reduce this national and international health issue. Thus, this systematic review and meta-analysis was conducted to evaluate the prevalence of NSIs in Iranian HCWs and to compare the results with those of national and international studies.

## Methods

### Search strategy

This systematic review and meta-analysis analyzed the prevalence of NSIs among the HCWs (nurses, midwives, doctors, and paramedics) in Iranian hospitals based on the articles published in national and international journals. National databases, (including Scientific Information Database (SID), MagIran, and IranMedex) and international databases, (including Google Scholar, Science Direct, PubMed, and Scopus) were searched to obtain the studies which conducted regarding the prevalence of NSIs. Articles were searched using the key terms percu* injur*, needle* stick injuries*, needlestick injur*, sharp* injur*, or Iran as well as all possible combinations of these terms. The Persian sites were also searched using the equivalent of these terms. Further, the sources of studied articles were reviewed to get access to other articles.

### Study selection and data extraction

First, a list of titles and abstracts was prepared from databases by two researchers independently. The inclusion criteria were as follows: (1) observational studies (cross-sectional, case-control, or cohort), (2) articles in Persian and English languages, and (3) methodological quality score ≥ 8. Qualitative studies, reviews, letters to editor as well as research conducted on students, dentists, and housekeeping staff were excluded from the study. The abstracts of articles were analyzed by two researchers based on the inclusion and exclusion criteria. The relevant articles were selected and their full texts were extracted. Each article was evaluated independently by two researchers. In the case of disagreement on selecting of an article, it was reviewed by a statistician who is an expert in meta-analysis study. A checklist was used to determine the quality of articles. It had been used in different meta-analyses by other researchers [17, 18].

This checklist consisted of 12 sections, including objectives, nature of intervention, methods, time period, sample size, sampling method, data collection, outcome variables, study population, cultural and linguistic range, and data analysis. Each section was scored from 0 to 1, and the range of scores for each article was between 0 and 12. Thus, articles which scored ≥ 8 were considered acceptable methodological quality. Moreover, we followed Meline (2006) seven steps recommendations for selecting studies to ensure quality of selected studies. First, we considered inclusion and exclusion criteria for the title and abstract. Secondly, we omitted studies that clearly meet one or more exclusion criteria. In the third step, we obtained the full text of the remaining studies. We evaluated the remaining studies for inclusion and exclusion in the next step. In step five, we included all studies that meet the inclusion criteria, but not exclusion criteria. Then, we excluded studies from study with reasons in the sixth step. Finally, we accepted some studies for our research in the last step [19]. Based on the inclusion and exclusion criteria, 44 qualified articles related to NSIs were selected from 2003 to 2016. Eventually, a prefabricated checklist was used to extract data from the selected articles. The checklist consisted of variables such as the corresponding author, publication year, study population, sample size, gender, the total prevalence of NSIs in each gender, type of questionnaire, region, and type of hospitals. All ethical issues were considered in conducting and reporting of this study.

### Statistical analysis

Since prevalence has binomial distribution, the prevalence variance was calculated by variance of the binomial distribution. Weighted average was used to combine the prevalence rates of various studies, and the weight allocated to every article was the inverse of the variance. Heterogeneity of data was evaluated by *I*^2^ index and Cochran’s *Q* test. Heterogeneity was classified into the following three categories: *I*^2^ index < 25% (low heterogeneity), *I*^2^ index = 25–75% (average heterogeneity), and *I*^2^ index > 75% (high heterogeneity). Considering the heterogeneity index (*I*^2^) which was more than 75% (97.6%), as well as the significance of the Cochran’s *Q* (*p* < 0.0001). Thus, random effects model was used to analyze data in this study. Meta-regression analysis was used to evaluate the association between the prevalence of NSIs and publication year and sample size in the selected studies. Also, subgroup analysis was used to assess the prevalence of NSIs for each gender, type of the hospital, instrument, and sampling methods. The role of each study on the final results was investigated using sensitivity analysis. Egger regression asymmetry test was used to evaluate the effects of small studies and publication bias. Data were analyzed by STATA (version 12) software.

## Results

**Fig. 1:**
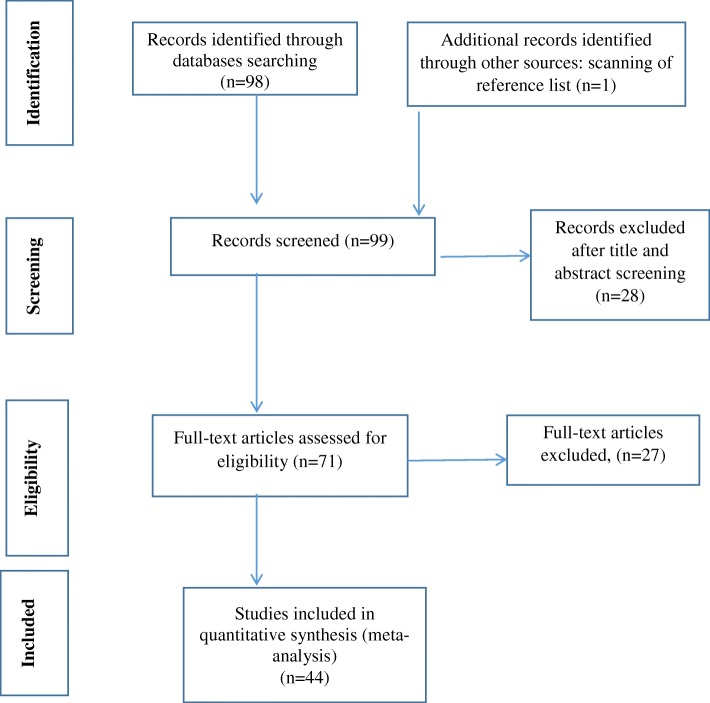
The process of surveying, screening, and selecting the articles for systematic review and meta-analysis based on PRISMA guideline. Ninety-nine studies were identified in the initial search (identification). After the title and abstract screening, 28 studies were excluded (screening). Based on the inclusion and exclusion criteria, 27 studies were excluded from the final analysis (Eligibility). A total of 44 articles were included in meta-analysis

All observational studies carried out on the prevalence of NSIs in Iran were evaluated without time limit and were subjected to systematic review and meta-analysis according to PRISMA guideline [20]. Ninety-nine studies were identified in the initial search. After the title and abstract screening, 28 studies were excluded. Based on the inclusion and exclusion criteria, 27 studies were excluded from the final analysis. A total of 44 articles were included in meta-analysis (Fig. 1).

A total of 44 articles [2, 5, 10, 21–62] were included in meta-analysis. The study sample included 16,105 samples with the mean of 366 samples in each study. The maximum and minimum sample sizes were found in studies conducted by Askarian (1555 samples) [54]and Mohammad Nejad and Hajivandi (each 68 samples) [22, 45]. The general characteristics of the selected studies are presented in Table 1.

**Table 1:** Characteristics of selected studies

First author (references)	Year	Sample size (*n*)	Area	Study population		Type of hospital	Total prevalence (%)
				All healthcare workers	Only nurses		
Geravandi [10]	2016	600	Ahvaz	*		Teaching	8.1
Jahangiri [23]	2016	168	Shiraz	*		Teaching	54
Salman zadeh [25]	2016	377	Dasht-e Azadegan	*		Teaching	18.4
Hajivandi [22]	2015	68	Bushehr		*	Teaching	58.8
Mahmoudi [61]	2015	100	Tehran		*	Marital	41
Izadi [26]	2015	09	Tehran	*		Teaching	26.9
Balouchi [21]	2015	240	Kerman		*	Teaching	39
Mirzaei-Alavijeh [24]	2014	70	Rafsanjan		*	Teaching	41.4
Ghanei Gheshlagh [2]	2014	120	Saghez		*	Teaching	44.2
Bijani [29]	2013	246	Qazvin		*	Teaching	31.3
Adib Hajbaghery [5]	2013	298	Kashan	*		Teaching	38.3
Rezaei [28]	2013	514	Tehran		*	Teaching	26
Shoghli [27]	2013	593	Zanjan	*		Teaching	26.3
Rezaei [32]	2012	991	Tehran	*		Teaching	16.8
Sharifian [31]	2012	350	Tehran	*		Teaching	19.7
Ghannad [35]	2012	89	Hamadan	*		Teaching	51.6
Nejadghaderi [33]	2012	186	Rafsanjan	*		Teaching	54.1
Tirgar [30]	2012	333	Babol		*	Teaching	59.7
Hashemi [34]	2012	700	Hamadan	*		Teaching	24.1
Ehsani [37]	2012	328	Tehran		*	Teaching	45.1
Mohammadi [38]	2011	138	Qazvin		*	Teaching	38.4
Bijani [36]	2011	172	Qazvin		*	Teaching	32
Rahnavard [39]	2011	500	Rasht		*	Teaching	77.2
Shiva [40]	2011	355	Tehran	*		Teaching	49.3
Khalooei [44]	2010	338	Kerman		*	Teaching	33
Nasiri [41]	2010	352	Sari	*		Teaching	75.6
Moradi [46]	2010	182	Bahar	*		Teaching	43.8
Heidari [43]	2010	77	Borujen	*		Teaching	74
Mohammadi Nejad [45]	2010	68	Tehran		*	Teaching	7
Gholami [42]	2010	400	Urmia	*		Teaching	26.8
Kazemi Galougahi [48]	2010	158	Tehran		*	Teaching	57
Mohammad Nejad [50]	2009	218	Tehran		*	Teaching	43.1
Rakhshani [62]	2009	231	Zahedan	*		Teaching	64.9
Abdi [47]	2009	298	Jahrom	*		Teaching	47.3
Joneidi Jafari [51]	2008	613	Tehran		*	Marital	32.7
Lotfi [52]	2008	0	Astara	*		Teaching	7
Azadi [53]	2007	111	Tehran		*	Teaching	45
Askarian [54]	2007	1555	Shiraz		*	Teaching	26.3
Ebrahimi [55]	2007	180	Shahrud		*	Teaching	63.3
Vahedi [57]	2006	847	Sanandaj	*		Teaching	64.9
Nazmieh [59]	2005	1020	Yazd	*		Teaching	37.8
Rahim nejad [56]	2005	434	Urmia	*		Teaching	52.5
Poorolajal [60]	2004	1000	Hamadan	*		Teaching	24
Hoseini Shokouh [58]	2003	88	Tehran	*		Marital	33

**Fig. 2:**
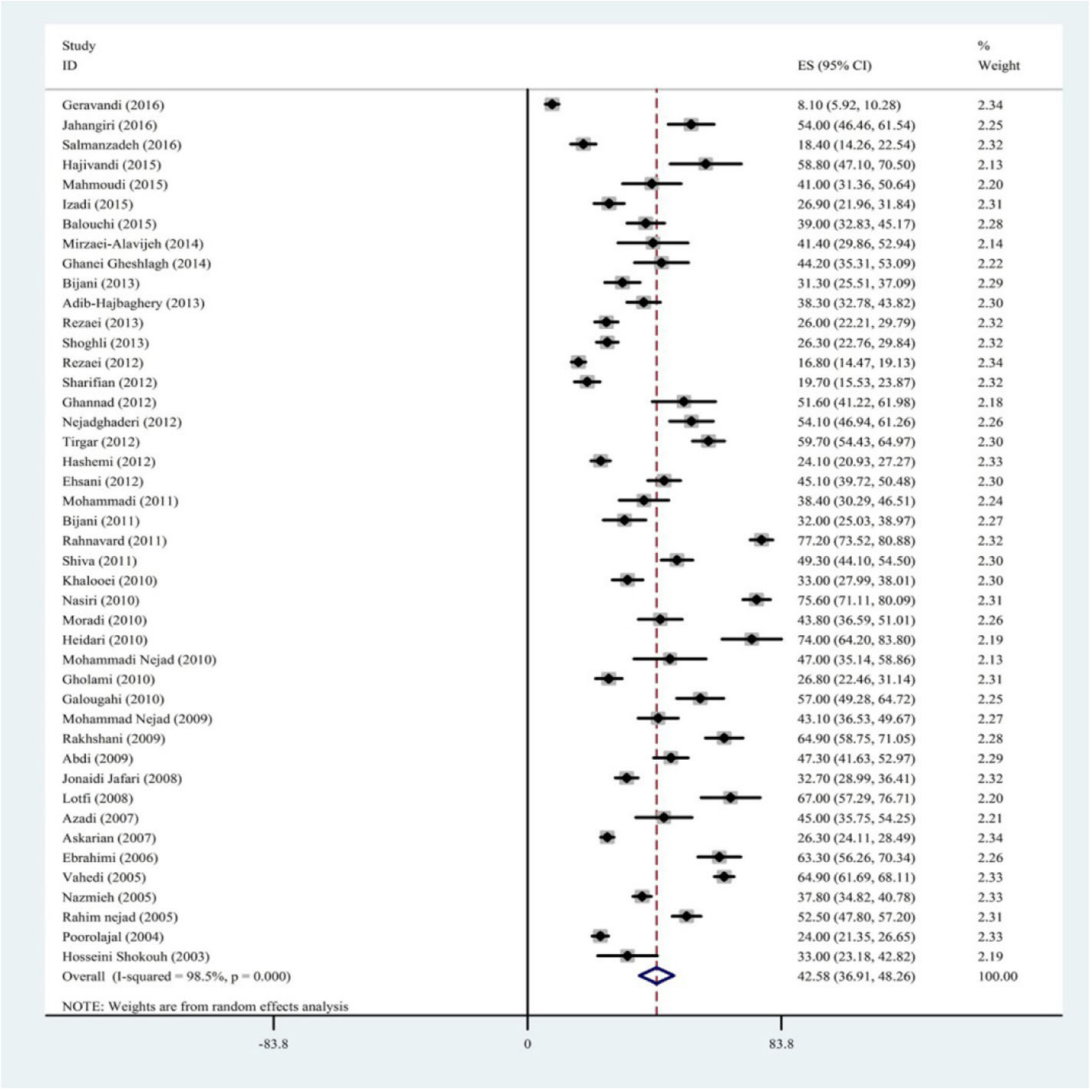
Prevalence of NSIs in HCWs according to the studies conducted in Iran. This figure demonstrates the information of every single study according to the year of studies conducted, the first author, and the final outcome of the studies. The random effects analysis has been used to estimate the overall prevalence as it is mentioned at the bottom of the figure. This figure displays the estimation of each study with square and the 95% confidence interval for the relevant estimation with a horizontal line. Square size shows the weight of each study in meta-analysis. The diamond shown at the bottom of the figure indicates the weight of all squares. Horizontal diameter of the diamond shows the possible range of prevalence outcome. Two vertical lines are shown in the figure. The dotted vertical line which is in line with diamond vertical axis shows the overall meta-analysis outcome (pooled prevalence). Another continuous vertical line shows the null hypothesis or no effect which is zero in prevalence, incidence, and means

The total prevalence of NSIs was 42.5% (95% CI 37–48) in this study. Since, the studies had been performed on either all HCWs (except students, dentists, and housekeeping staff) of hospitals or exclusively on nurses. Therefore, the prevalence rate was analyzed separately for either nurses or all other health care groups. Findings showed the prevalence of NSIs was more in nurses than in other health care groups (44% vs. 41%). The prevalence of NSIs was reported for each gender separately. Findings showed the prevalence of NSIs was more in women than in men (47% vs. 42%) [2, 10, 23, 27, 28, 30, 32, 36, 42, 55, 56]. Result revealed that the prevalence of NSIs was more in male nurses (49%; 95% CI 28–70) compared to men in other health care groups (37%; 95% CI 18–56). It was also more in female nurses (47%; 95% CI 31–61) compared to women in other health care groups (46%; 95% CI 36–58) (Fig. 2).

The prevalence of NSIs for each hospital showed the prevalence rate was more in teaching hospitals than in military hospitals (43% vs. 34%). More details on the prevalence of NSIs for subgroups are presented in Table 2.

**Table 2:** Prevalence of needlestick injuries in each subgroup

Variables	Groups	Number of studies	Sample size	Prevalence	95% confidence interval	Heterogeneity		
						P	*Q*	I^2^
Gender	Male	11	1571	42	26–58	98.4	619.07	0.0001
	Female	11	2609	47	36–58	97.4	380.2	0.0001
Tool	Researcher-made	39	12,780	43	36–50	98.6	2769.03	0.0001
	Other	5	3325	36	29–43	93.7	63.55	0.0001
Sampling method	Random	9	3231	42	29–55	98.4	506.97	0.0001
	Census	15	5835	43	34–52	98.3	840	0.0001
	Other	6	795	50	38–62	92.1	63.09	0.0001
	Unknown	14	6244	39	29–48	98.8	1090.25	0.0001
Type of hospital	Teaching	41	14,392	43	37–49	98.6	2917.03	0.0001
	Military	3	1713	34	30–38	20	2.50	0.286

**Fig. 3:**
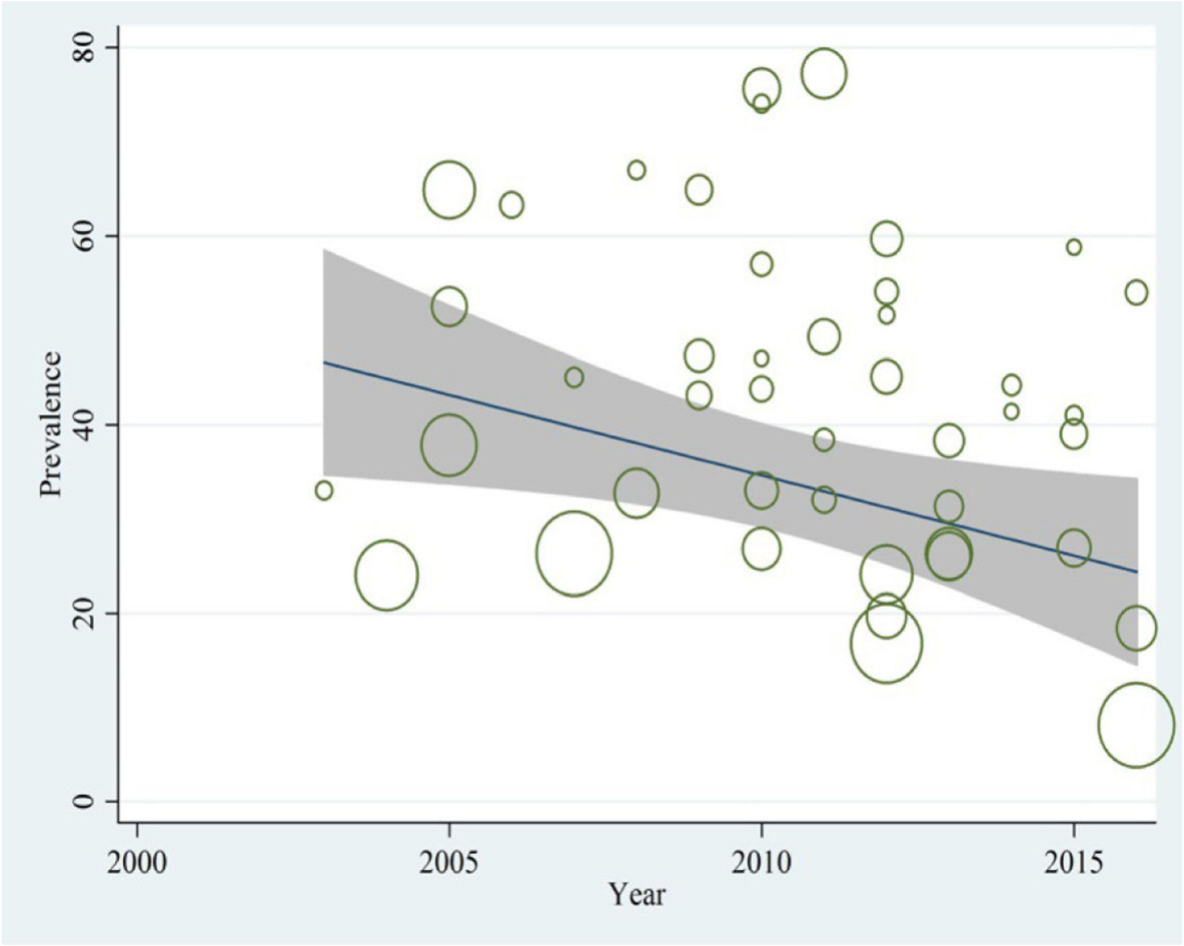
Reduction in prevalence of NSIs during 2003–2016 according to meta-regression. This figure shows meta-regression analysis of needlestick prevalence based on selected studies’ publication years. The vertical axis represents the prevalence, and the horizontal axis represents the selected studies’ publication year. Slope of the regression line indicates an increase or decrease of study effect using REML estimation. Given the slope of the regression line is descending in this figure, it can be inferred that as the studies’ publication year has been increased, the prevalence of needlestick has been decreased. Gray color lines around the slope of the regression line indicate 95% confidence interval. Each circle demonstrates one selected study and the size of each circle corresponds to the weight assigned to each study. Reverse weight corresponds to the standard error of each study

**Fig. 4:**
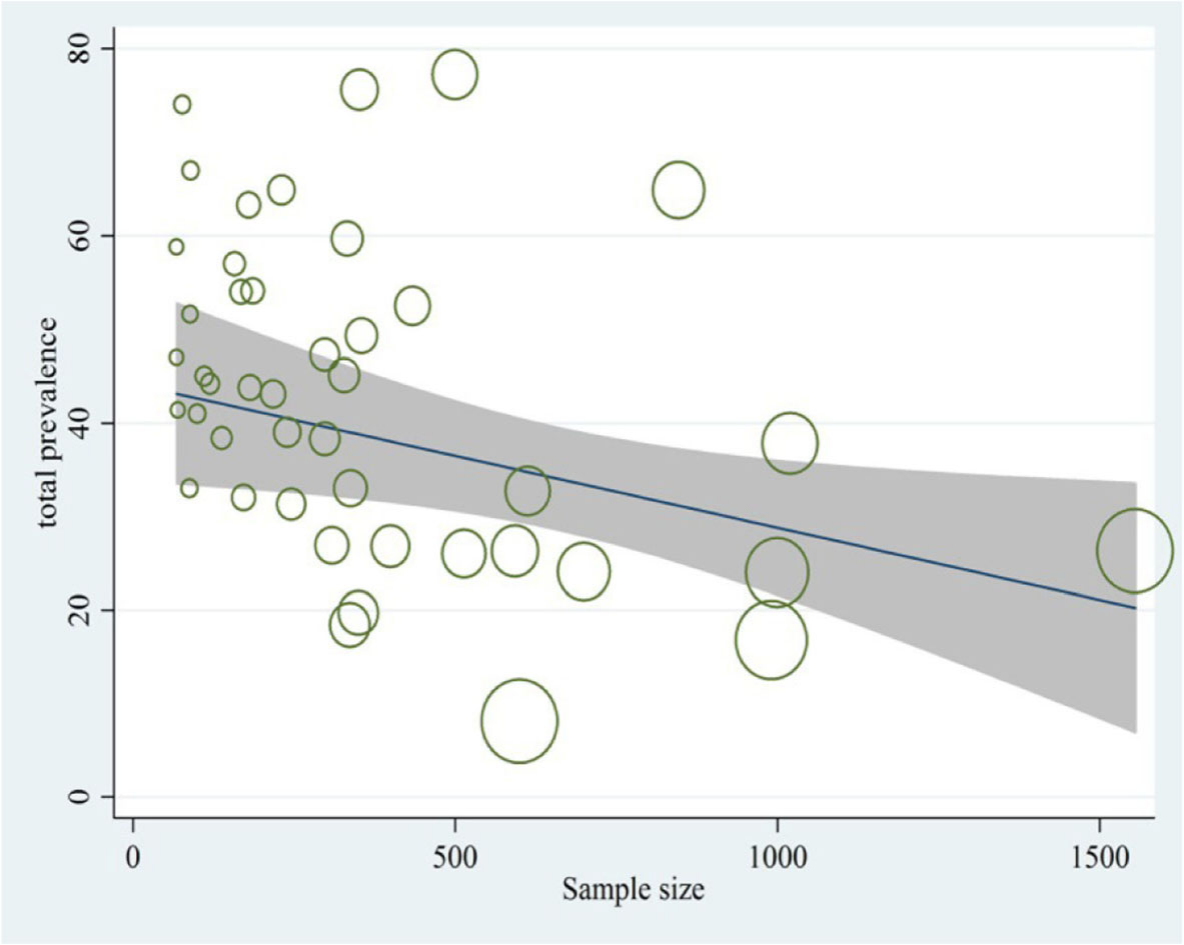
Total prevalence of NSIs based on the sample size of selected studies by meta-regression analysis. Circles show the weight of the studies. The figure indicates a significant association between the prevalence of NSIs and sample size. The prevalence rate was reduced significantly with a rise in sample size

The results of meta-regression analysis showed no significant relationship between the prevalence of NSIs and publication year (*p* = 0.141( (Fig. 3). However, there was a significant association between the prevalence of NSIs and sample size. The prevalence rate was reduced significantly with a rise in sample size (*p* = 0.011) (Fig. 4).

**Fig. 5:**
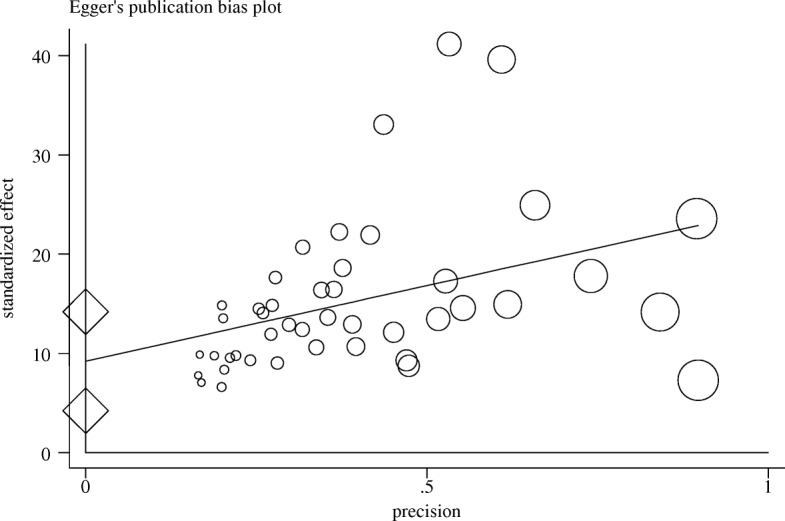
Publication bias. This figure is used to investigate the publication bias of studies. Circles show selected studies, and the area of each circle is equivalent to the weight of each study. The horizontal axis represents accuracy, and the vertical axis represents the standardized effect. The line shown in this figure is a regression line related to Egger’s regression test. It shows that whether this line cut the vertical axis at the point near zero or not. If this line distance from zero, it indicates a bias in publishing the results. If there is no publication bias, it is expected that this line passes from origin to a point near the origin. Since intercept (width from origin) is close to 9 in this figure, we conclude that publication bias is significant. Two diamonds that are plotted on the vertical axis indicate confidence interval corresponds to the coefficient obtained from the regression test β_1 that it is 4.2 to 14.1. Because zero is not included in the confidence interval. Therefore, it could be concluded that the publication bias is significant

Further, the findings revealed that publication bias was significant in this study (*p* = 0.001) (Fig. 5). The results of sensitivity analysis indicated that absence of every single study did not make a significant change in frequency estimates. On the other hand, none of the studies had a significant effect individually on estimating the pooled prevalence of NSIs.

## Discussion

Numerous studies on the prevalence of NSIs have reported different results. In spite of all precautions, NSIs are inevitable. This study showed the prevalence of NSIs was 42.5% in HCWs in Iran, which was higher than the prevalence rate in Turkey (30.1%) and Qatar (20.9%) [14, 63] and less than Jordan (91.8%) and Pakistan (94%) [64, 65]. Prevalence of NSIs varies depending on hospital conditions and standards, overcrowding of patients, hospital ward, type of health care personnel, and their skills. Context, culture, and access to resources are the main reasons for the difference in the prevalence of NSIs in these societies. Types of hospital policies, rules and regulation, and the way infection control nurses taking hard on the staff make many of the employees never report their injuries. On the other hand, many personnel get confused and do not know where to report and what forms to complete after NSIs due to rapid changes in the hospitals’ guidelines and policies [66]. Dissatisfaction with follow-up by administrators after reporting the events, low risk perception, and time-consuming protocol [66, 67] are some other reasons for underreporting NSIs. Therefore, employees prefer not to report their injuries. Thus, this will cause the validity of existing data to be disrupted to some extent.

In general, half of the HCWs experience NSIs during their working career [68]. However, NSIs have not been reported by victims in many cases due to various reasons previously listed. Thus, the actual rate of NSIs may be underestimated. In the present study, the prevalence of NSIs was reported to be 42%, which was reduced by 16% compared to the results of the study by Sayehmiri et al. on the prevalence of NSIs in Iran [69]. This reduction in the prevalence rate could be due to underreporting of NSIs, which may be associated with the time-consuming nature of reporting the injuries, believing in the low risk of NSIs for transmission disease, being unwilling or lacking the time for follow-ups and treatment [70]. Because of the high prevalence of NSIs, there have been efforts to provide more safety training, awareness, and workshops. And because of better knowledge among HCWs, there is reduction in prevalence. This could be one of the reasons for reducing of the prevalence in our study. Several studies reported the necessity of holding educational class in order to prevent NSIs and reduce the prevalence of NSIs [26, 69].

Further, the findings indicated that the prevalence of NSIs was more in women compare to men. Since the number of female healthcare workers is several times higher than males in Iranian hospitals [26], female nurses have more responsibilities than male nurses [63] and women are more likely to be stressed than men [71]. A study showed that HCWs who had job-related stress were 7.3 times more likely to face NSIs [72]. This finding is in line with the results of studies conducted in Ethiopia and Saudi Arabia [73, 74]. The study of Shah et al. showed women suffered from NSIs twice more than men [63]. Kebede et al. reported that half of NSIs occurred in women [75]. Similar to our result, recent studies showed that women are more likely to report injuries, follow the tests and post-traumatic care compared to men because of feeling pain or worrying about bloodborn infectious diseases following NSIs [2, 55].

Moreover, the prevalence of NSIs was more in nurses than in other HCWs, confirming the results of studies carried out in India and Georgia [76, 77]. Similar to our findings, the systematic review of Khraisa et al. showed the prevalence of NSIs was higher in nurses than other HCWs in hospitals (64% vs. 44%) [78]. A study conducted in Jordan reported the maximum and minimum prevalence rates of NSIs in nurses (81%) and midwives (1%) [64]. The findings of a study in Pakistan showed the female nurses have a higher prevalence rate of NSIs than other professionals [79]. In the research carried out by Yoshikawa et al., NSIs occurred in nurses three times more than other HCWs [80]. In line with our findings, a study conducted in Portugal by Martins et al. reported the most NSIs occurred in female nurses compared to other HCWs [81]. Similar to other study results, our findings indicate high prevalence of NSIs in nurses is due to high workload, increased sharp objects exposure, inadequate staffing, and long working hours [11, 13, 75]. In addition, our findings indicate that the prevalence of NSIs was higher at teaching hospitals than military hospitals. This may be due to overcrowding and understaffed shifts, or better reporting of NSIs, or special policies of hospital management in these regions and these types of hospitals.

Reviewing literature shows that the incident of NSIs is associated with three main factors: engineering (the form of devices), organizational (injury reporting policies), and behavioral (recapping needles and disposing of them) factors [13]. A review of the literature showed that those three factors have not mentioned clearly in studies conducted in Iran. However, according to the results, the cause of most injuries was reported to be behavioral factors such as recapping the needle [10, 69]. Our results indicated behavioral factors play important role in our health care settings. In spite of frequent education, many of the staff still are insistent on recapping needles. Unfortunately, this behavior remains the main cause of many NSIs. Various studies have reported that recapping has been the most common cause of NSIs in Iranian hospitals [10, 69].Thus, teaching safe injection methods [23] as well as correct use and disposal of sharp objects (standard precautions), safety-engineered device (SED) [13, 16, 23], which involves replacing conventional needles with safe needles, and teaching the correct use of safe needles have been proposed as the most important strategies to decrease the incidence and prevalence of NSIs. Adams believes that use of safe-engineered devices is more effective to reduce NSIs than developing policies, regular training of personnel and the use of personal protective equipment [82]. According to our clinical experience, new hospital guidelines and policies are presented more often to the staff in this regard, while the safe-engineered devices and personal protective equipment are not sufficiently available to personnel.

Some limitations of the current study included inadequate information of some articles, irregular distribution of studies around the country, small sample size, and unknown sampling method of some studies. Reporting an accurate estimate of this problem in Iran and comparing with other countries via meta-analysis highly recommended. We also suggest further studies to be conducted to investigate and compare the prevalence of NSIs in dentists, nursing and medical students, and housekeeping staff with other HCWs.

## Conclusions

The results of the present study indicated a relatively high frequency of NSIs. Non-compliance with specific standards on using of equipment, wearing protective devices, and disposing of sharp objects can play a pivotal role in increasing the possible risk of NSIs in the HCWs. Needlestick and sharps injuries can be reduced by taking such measures as supplying standard and safe equipment, holding training workshops regarding safety issues at work environment, providing enough staffing, and cutting down working hours.

### Implication for practice

Considering the high prevalence of NSIs among the Iranian HCWs, hospital managers are advised to introduce and adopt restructured guidelines and to supply safe needles and instruments for employees. They also need to develop and implement preventive programs such as installing safety enhanced devices [14, 21] in health sectors like needle cutter machines and adequate safety disposal boxes [2, 23]. Infection control managers/nurses need to hold training programs [23] focused on new methods of using sharp objects safely, observe safety principles and standards, and reinforce the practical skills of personnel. Because more than half of HCWs in Iran do not report their NSIs and expose themselves to the harmful consequences. Thus, nursing managers must try to break this culture of silence [8] with proper actions such as following up the incident seriously, facilitating the reporting process [2], and taking easy on the employees who receive these injuries. They also need to pay more attention to reporting and improving occupational behaviors such as avoiding recapping the needles [54] in order to decrease the incidence of NSIs and consequently reduce blood-transmitted infectious diseases. Nurse education specialists can provide educational activities to personnel to improve the knowledge and skills necessary to deal with this problem by different methods such as seminars, informative educational boards, pamphlets, and workshops [5, 22, 79]. Also, nursing researchers and policy-makers may develop standard tools like national surveillance for reporting NSIs in the entire country to help injured personnel’s and victims’ private information remains confidential in order to avoid social stigma.

## Abbreviations

BBPs: Bloodborne pathogens
HCWs: Health care workers
NSIs: Needlestick and sharps injuries
